# Suicidal behavior in persons attended in out-of-hospital emergency services in Spain

**DOI:** 10.3389/fpsyt.2023.1235583

**Published:** 2023-08-16

**Authors:** Javier Ramos-Martín, Carlos Gómez Sánchez-Lafuente, Ana I. Martínez-García, Pilar Castillo-Jiménez, José Guzmán-Parra, Berta Moreno-Küstner

**Affiliations:** ^1^Departamento de Personalidad, Evaluación y Tratamiento Psicológico, Universidad de Málaga, Málaga, Spain; ^2^Instituto de Investigación Biomedicina de Málaga (IBIMA), Málaga, Spain; ^3^Unidad de Gestión Clínica de Salud Mental, Hospital Regional Universitario de Málaga, Málaga, Spain; ^4^Unidad de Gestión Clínica (UGC) del Servicio de Urgencias de Atención Primaria (SUAP) del Distrito Sanitario Málaga, Málaga, Spain; ^5^Grupo Andaluz de Investigación Psicosocial (GAP) (CTS-945), Málaga, Spain

**Keywords:** suicide, out-of-hospital emergency department, suicide attempt, suicidal behavior, hospital contact, risk factors

## Abstract

**Background:**

The aims of this study were to describe the use of health services by patients attended for suicidal behavior by out-of-hospital emergency services and to identify the variables associated with the repetition of this behavior in Spain.

**Methods:**

An analytical, observational, retrospective study was carried out. A total of 554 patients attended by the mobile teams of the Primary Care Emergency (mt-PCES) of the Malaga Health District (Spain), after being coordinated by the 061 Emergency Coordination Center (ECC) were analyzed.

**Results:**

Of the total, 61.9% of the patients were women and the mean age was 43.5 years. Ninety-six percent (*N* = 532) of the patients attended by mt-PCES were transferred to hospital emergency services. Regarding clinical decision, of those transferred 436 persons (82%) were discharged home. Of the total sample 25.5% (*N* = 141) were referred to primary care, while 69% (*N* = 382) were referred to outpatient mental health care. Regarding follow up in the 6 months after being seen by emergency services, among those referred to a mental health facility, 64.4% (*N* = 246) attended the follow-up appointment while out of the total sample only 50.5% (*N* = 280) attended a follow-up appointment with an outpatient mental health service. Finally, it should be noted that 23.3% presented a relapse of suicidal behavior in the 6 months following index episode. The variables associated with repetition of suicidal behavior were older age, greater number of previous suicide attempts and having any contact with mental health services in the following 6 months.

**Conclusion:**

We believe that selective suicide prevention initiatives should be designed to target the population at risk of suicide, especially those receiving both out-of-hospital and in-hospital emergency services.

## Background

Suicide is one of the leading causes of death worldwide and is a major public health problem. The World Health Organization (WHO) indicated, in the latest report published, that annually more than 700,000 suicides occur globally, which means that one in every 100 deaths is due to suicide (1). In Spain, suicide continues to be the leading cause of out-of-hospital death, with 4,003 suicides in 2021, representing the year with the highest number of suicides recorded in the history of Spain, which corresponds to a rate of 8.5 suicides per 100,000 inhabitants (2).

These data, however, do not reflect the figure for nonlethal suicidal behavior (suicide ideation, planning and attempt) which is much higher. According to the WHO, worldwide, for every person who commits suicide, there are 20 who attempt suicide (3). In Europe, a recent systematic review conducted with epidemiological studies in the general population indicated that the annual prevalence of suicidal ideation, planning and attempted suicide was 3.6, 1.6, and 0.5%, respectively (4). In Andalusia, a population-based study yielded a point prevalence rate of 2.4% for suicide ideation, 1.1% for suicide planning and 0.6% for suicide attempts (5).

While population-based studies are important to understand the numbers of nonlethal suicidal behavior in the general population, those based on the population being treated by health services provide information on those individuals presenting with more severe suicidal behaviors. In addition, high use of both primary care, specialist and emergency services is observed in the weeks prior to suicide (6–8). Specifically, those studies carried out in the emergency services setting, both in-hospital and out-of-hospital, are of great use for analyzing suicide, as they are the main care facilities where people with suicidal behavior are treated (9). Moreover, this demand for care tends to increase in the weeks prior to death (10), and therefore these contacts represent a potential opportunity for suicide prevention, through the identification of people at risk of suicide and the availability of treatment or interventions more appropriate to their needs (11).

Regarding the variables associated with the repetition of suicidal behavior, Mirkovic et al. (12), found that among the risk factors for relapse in suicide attempts were seeking psychiatric care with medication and the presence of a history of suicide attempts. On the other hand, De Santiago-Díaz et al. (13), affirms that the repetition of suicide attempts is frequent even when the person is being followed up by a mental health service. In addition, this study shows that people who present suicidal behavior tend to repeatedly use emergency services for other psychiatric and medical reasons, indicating that the conventional approach to comorbidity with suicidal behavior is insufficient. Only having a mental health problem was significantly related to repeated suicidal behavior at 6-months follow-up.

The study of Suárez-Pinilla et al. (14), found that seeking emergency psychiatric help for problems other than suicidal behavior during follow-up was a predictive factor of both suicide attempts and suicidal ideation. They also found that a history of suicide attempts as well as contact with psychiatric outpatient units during follow-up predicted both general suicidal behavior and suicide attempts in particular.

Our line of research has focused on studies of suicidal behavioral calls to out-of-hospital emergency services settings only (9, 15, 16). There are also several studies focused on this population in the hospital emergency setting (17–19). However, as far as we know, there is no studies focus on both out and in emergency services. With this study we intended to go a step further to understand the continuity of care of these patients when they are transferred by out-of-hospital emergency teams to the hospital.

The present study aimed to describe the use of health services by patients attended in out-of-hospital emergency services for suicidal behavior and to identify the variables associated with the repetition of this behavior.

## Methodology

### Design and scope of study

This was an analytical, observational, retrospective study based on the information recorded in the medical records.

The study population comprised the individuals registered in the database of the 061 Emergency Coordination Center (ECC) of Malaga (Spain) who were attended by the mobile teams of the Primary Care Emergency (mt-PCES) of the Malaga Health District. This Health District covers a population of 620,889 inhabitants (year 2018) and belongs to the Andalusian Public Health Service, which provides universal health coverage to anyone living in the region. In addition, at the hospital level, this district includes the two public hospitals in the province of Malaga (Regional University Hospital of Malaga and Virgen de la Victoria University Hospital). This study analyzed the calls to 061 ECC between the years 2018 and 2020.

The 061 ECC is located in each of the eight provinces of the Autonomous Community of Andalusia (southern Spain). Its function is to coordinate and register the urgent healthcare requests received by any emergency telephone (061, 112). Once the telephone call has been answered, the operator and/or the physician record all the information about the event in order to choose the best available resource (ambulance, mobile intensive care unit, helicopter, etc.) depending on the reason and priority of the request. If deemed necessary, a mt-PCES of the Malaga Health District travels to the site of the event to attend to the affected individual *in situ* (20). After completing the evaluation, out-of-hospital emergency doctors can discharge the patient with a treatment plan that includes patient care by a mental health center or transfer the patient to in-hospital emergency services if they need urgent attention.

Once patients with suicidal behavior are transferred to in-hospital emergency services, they are first seen by a nursing professional who establishes a 5-level priority status based on the Spanish Triage System. Subsequently, an emergency physician performs an evaluation and examination by organs and systems of the person and requests complementary tests depending on the suspected diagnosis. Based on the findings of this evaluation, a clinical decision is made, which may be discharge home with a treatment plan or referral of the patient to a psychiatrist. The psychiatrist, in turn, may establish a clinical decision to discharge the patient home with a treatment and follow-up plan or admit the patient to hospital. In cases of extreme severity, requiring intensive care unit measures, psychiatric care is provided at a later stage when their physical symptoms have stabilized.

### Sample and selection procedure

The sample for this study comprised people who engaged in suicidal behavior and requested care from the 061 ECC and were attended by the mt-PCES of the Malaga Health District (Spain).

A case was considered to be suicidal behavior and to be included in this study if any of the following criteria were met: (a) when out-of-hospital emergency doctors attending the patient at the scene of the event makes a clinical judgment, based on ICD-9 (21), with the codes: V62.84 (suicidal ideation) or E950-E959 (suicide and self-inflicted injuries by hanging, drowning, falling, etc.), regardless of the classification of the operator. Consequently, in order not to miss any patient with suicidal behavior, we have also included cases that met the following criterion: (b) when the 061 ECC assigns a code according to its own classification referring to suicide (tendency to self-injury and suicide; threats of suicide; suicidal ideation; included in the Psychiatric category) and, in addition, out-of-hospital emergency doctors attending the patient at the scene makes a clinical judgment, based on ICD-9 (21), with any code related to poisoning since is the one of the main methods used in previous suicide attempts.

A person could make more than one call during the study period, so we decided to analyze the sociodemographic characteristics of the first episode to avoid biases.

### Variables

The sources of information for this study were the electronic medical records, in which all contacts that patients have had with public health services (Primary Care, Specialized Care and Hospital and Outpatient Emergency Services) are recorded.

Data collection was carried out by two co-authors of the article (JR and CGSF), who performed a comprehensive consultation of the electronic medical records of the persons included in the study to extract information on the variables of interest. The search was carried out in April 2022.

The dependent variable of the study was the repetition of any act or thought of suicide that the person performs and that is evaluated in a health service or ends in death by suicide during the 6 months following discharge from hospital emergency services, categorized dichotomously (yes/no).

#### Independent variables

Once the calls for suicidal behavior were identified at the 061 ECC, information was collected for the following variables. (1) Sex; (2) Age; (3) Active mental health problems recorded in primary care medical records by physician according to ICD-9; (4) Number of previous suicide attempts in the patient’s life attended by public emergency health services (before to be included in the study); (5) Any contact with a public mental health facility in the 6 months prior to the call (yes/no); (6) Number of episodes of suicidal behavior requiring urgent care in the 6 months prior to the call; (7) Whether the patient had been transferred to in-hospital emergency care by mt-PCES (yes/no); (8) Patient care by psychiatry in in-hospital emergencies (yes/no); (9) Clinical decision after emergency care (admission/discharge to home/no decision due to patient absconding) and (10) Care facility to which the patient was referred (primary care/mental health); (11) Any contact with a public mental health facility in the 6 months following the request (yes/no). Finally, we examined whether the patient’s death and the cause of death had been recorded in his or her medical record up to the date of data collection (April 2022).

The study complied with the ethical criteria for research and was approved by the Ethics and Research Committee of Northeastern Malaga.

### Statistical analysis

First, the sample was described in terms of the number of cases and percentage for qualitative variables and the mean, standard deviation, or, in the case of variables that did not meet normality criteria, median and interquartile range for quantitative variables.

A bivariate analysis was then performed to describe the sample in terms of independent variables using Student’s *t*-test or the Wilcoxon-Mann–Whitney test for continuous variables according to their distribution, and the chi-square test for categorical variables. Subsequently, a stepwise logistic regression was performed using the forward elimination model to determine the factors that influenced the repetition of suicidal behavior by introducing into the model the variables with a *p* value ≤ 0.20 from the previous analysis. The continuous independent variables were previously analyzed using the Box-Tidwell test to verify that there was no violation of the logit linearity principle. The Hosmer-Lemeshow test was used to check the goodness-of-fit of the model. A significance level of 0.05 was established for all analyses.

SPSS Statistic version 25.0 was used to perform the statistical analyses.

## Results

### Description of the sample

**Figure 1:**
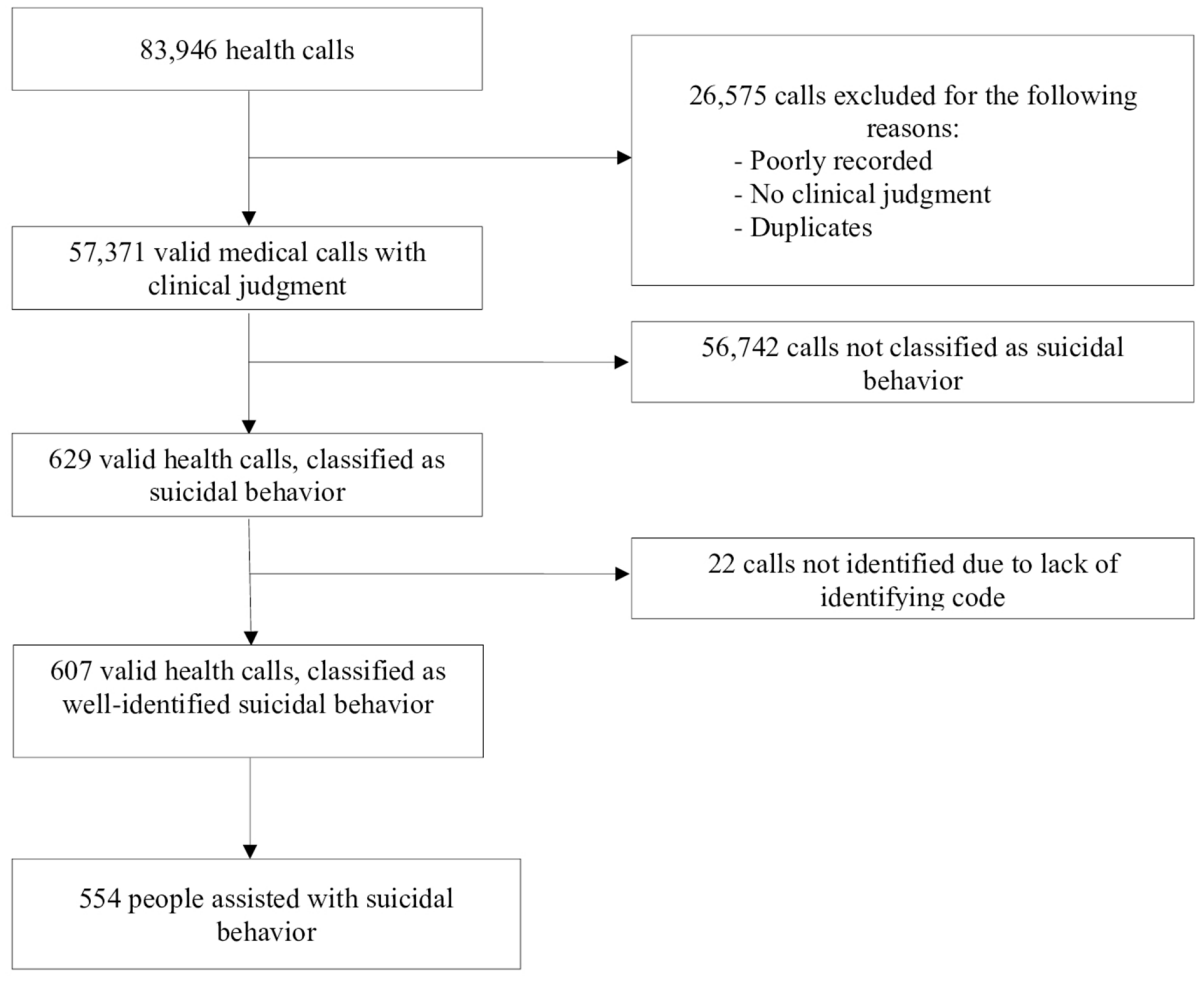
Flow chart of the selection process of the sample selected at the 061 Emergency Coordination Center.

The initial database had 83,946 health calls for any reason made to the 061 ECC during the years 2018–2020, corresponding to the Malaga Health District. For this study, we first excluded all calls that were poorly recorded, did not have a clinical judgment assigned by the health professional attending the patient on site or were duplicate records (*n* = 26,575). Subsequently, calls that were not identified as suicidal behavior were excluded (*n* = 56,742). The total number of valid health calls classified as suicidal behavior was 629 (0.7%). Twenty-two calls were excluded because they lacked an identification code to locate the digitalized clinical history, leaving a total of 607 valid health calls classified as suicidal behavior and identified in electronic medical records, which corresponded to a sample for this study of 554 persons attended by 061 ECC healthcare workers of the Malaga Health District for suicidal behavior (Figure 1).

The sociodemographic results of the overall sample (Table 1) showed an unequal distribution in terms of sex, with more women (61.9%) than men (38.1%) attended. The mean age at the time of call was 43.5 years. Analyzing by age group, the largest number of people were aged 45–59 years (34.1%), followed by those aged 30–44 years (28.7%), 15–29 years (21.5%), and over 60 years (14.8%).

**Table 1:** Distribution of the variables of the persons attended for suicidal behavior in the 061 Emergency Coordination Center (*n* = 554) according to repetition of suicidal behavior in the 6 months following the call to the 061 Emergency Coordination Center.

Variables	Total (*N* = 554)	Not repeated (*N* = 425)	Repeated (*N* = 129)	*p*
**Sociodemographics**				
Sex (female), *N* (%)	343 (61.9)	269 (63.3)	74 (57.4)	0.22a
Age, mean (SD)	43.54 (16.07)	44.39 (16.48)	40.74 (14.33)	0.11b
Active mental health problems, *N* (%)				**<0.001a**
No active problems	236 (42.6)	196 (46.1)	40 (31)	
1	185 (33.4)	145 (34.1)	40 (31)	
2	102 (18.4)	68 (16)	34 (26.4)	
≥3	31 (5.6)	16 (3.8)	15 (11.6)	
Number of previous suicide attempts*				**<0.001a**
0	245 (44.2)	241 (56.7)	4 (3.1)	
1	83 (15)	67 (15.8)	16 (12.4)	
Between 2 and 4	127 (22.9)	78 (18.4)	49 (38)	
Between 5 and 9	64 (11.6)	29 (6.8)	35 (27.1)	
More than 9	35 (6.3)	10 (2.3)	25 (19.4)	
Any contact with public mental health facility in 6 months prior to call, *N* (%)	223 (40.3)	146 (34.4)	77 (59.7)	**<0.001a**
Number of episodes of suicidal behavior requiring urgent care in the previous 6 months, median (IQR)	0 (0)	0 (0)	0 (1)	**<0.001c**
**Aftercare**				
Transfer to in-hospital emergency care, *N* (%)				0.28a
Yes	532 (96)	406 (95.5)	126 (97.7)	
No	22 (4)	19 (4.5)	3 (2.3)	
Psychiatric care in in-hospital emergencies, *N* (%)**	423 (76.4)	320 (75.3)	103 (79.8)	0.29a
Clinical decision, *N* (%)***				0.33a
Admission to a hospital ward	65 (12.2)	46 (10.8)	19 (14.7)	
Discharge to home	436 (82)	357 (84)	101 (78.3)	
No decision (absconded)	31 (5.8)	22 (5.2)	9 (7)	
Referral after urgent care, *N* (%)				**0.002a**
Mental health	382 (69)	279 (65.6)	103 (79.8)	
Primary care	141 (25.4)	124 (29.2)	17 (13.2)	
No referral (absconded)	31 (5.6)	22 (5.2)	9 (7)	
Any contact with public mental health facilities in the 6 months following the request, *N* (%)	280 (50.5)	185 (43.5)	95 (73.6)	**<0.001a**

a: Chi-square test; b: Student’s *t*-test; c: Wilcoxon’s Mann–Whitney test.

* Number of suicide attempts in the patient’s life that led to attending public emergency health services. This not included the suicide attempt that leads to being included in the study.

** Persons who were treated by a psychiatrist upon transfer to the hospital.

*** The 22 cases that were not transferred to the hospital are not included. Thus *N* = 532.

Values in bold represent statistically significant values as opposed to those not highlighted in bold which are not significant.

Regarding the active mental health problems recorded in the primary care medical record at the time of the self-injurious behavior, 236 persons (42.6%) had none recorded. Conversely, 318 persons (57.4%) did have active mental health problems recorded in the Primary Care medical record, of whom 102 (18.4%) had two comorbid mental health problems, and 31 (5.6%) had 3 or more comorbid mental health problems. Among the most frequent mental health problems were anxiety disorders (24.7%), followed by unipolar depressive disorders (24%), and personality disorders (11.4%).

Regarding the number of previous suicide attempts attended in a public health service, no suicide attempts were found in 245 persons (44.2%), 83 (15%) made one attempt, 127 (22.9%) made between 2 and 4 attempts, 64 (11.6%) made between 5 and 9 attempts, and 35 (6.3%) made more than 9 suicide attempts.

From the information consulted in electronic medical records, 13 people had died by the time the data were reviewed, of which only one case (0.2%) was directly caused by a suicide attempt (ingestion of caustics). The remaining cases died from aging-related processes or organic diseases (cancer and cardiovascular disease).

### Description of health care

**Figure 2:**
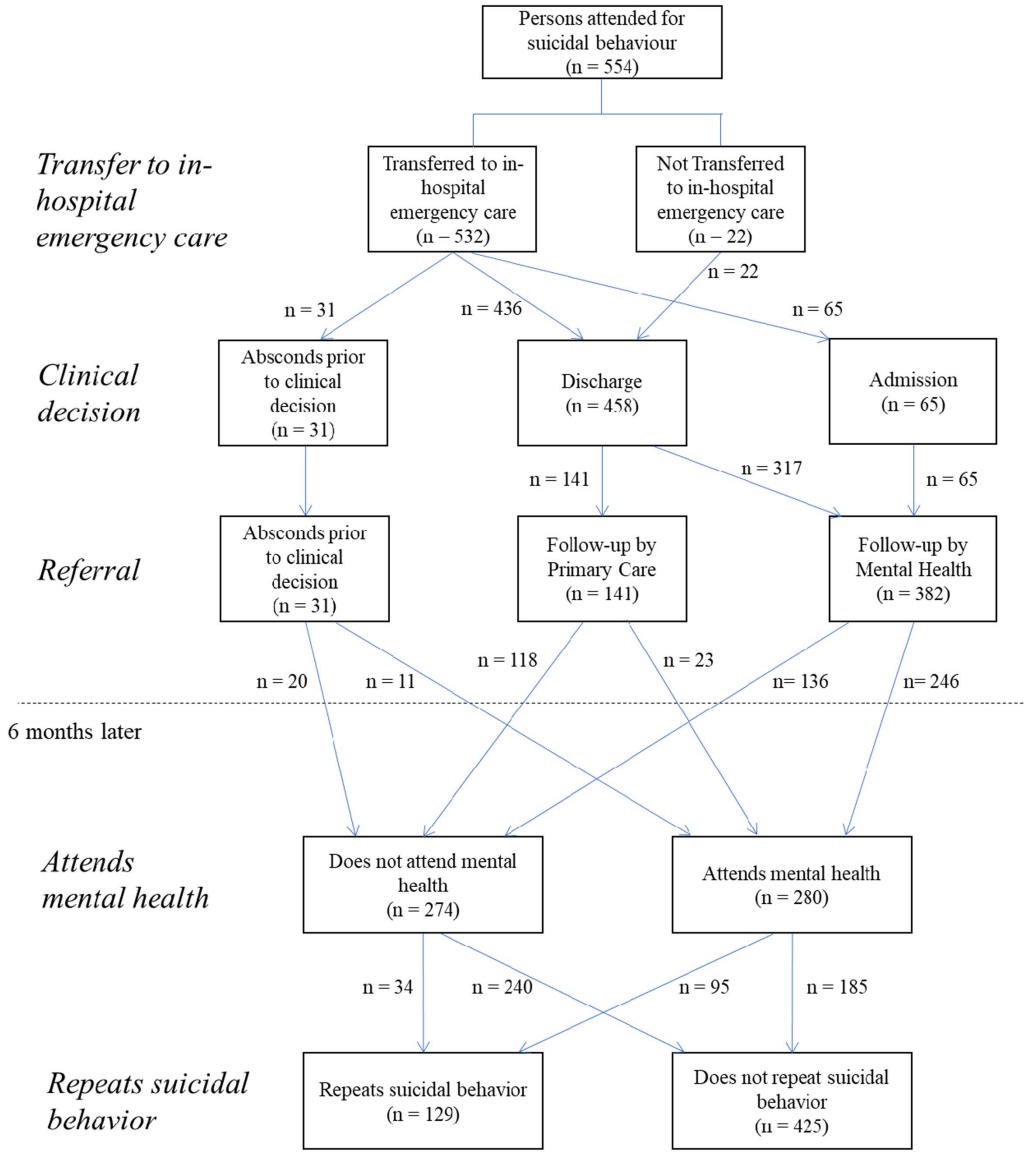
Flow chart of the care pathway of the persons attended by the 061 Emergency Coordination Center for suicidal behavior during the 6 months following the call.

Of the total sample attended by mt-PCES of the Malaga Health District (*n* = 554), 532 persons (96%) were transferred to in-hospital emergency services; while 22 persons (4%) were not transferred to hospital as they were discharged by the out-of-hospital emergency doctor. Of those transferred, 501 (94.2%) completed the urgent care circuit, while in 31 cases (5.8%) the person left the hospital emergency department of their own free will before the clinical decision was made (Figure 2). Overall, 423 people (76.4%) were seen by a psychiatrist.

Regarding the clinical decision of patient transferred to hospital (*N* = 532), 436 cases (82%) were discharged home, while 65 cases (12.2%) were admitted to the hospital ward and subsequently referred to an outpatient mental health unit. The mental health inpatient unit was the most frequent option among those admitted (55; 84.6%), followed by internal medicine (4; 6.1%) and intensive care (4; 6.1%). Among the total sample whose clinical decision was discharge home (*n* = 458, 82.7%), 141 cases (30.7%) were referred for follow-up in primary care, while 317 (69.2%) were referred for follow-up in an outpatient mental health facility. The total number of patients scheduled for mental health follow-up was 382 (69% of the total sample) (Figure 2).

In the 6 months follow up after being seen by emergency services among the 382 persons referred to a mental health unit, 246 (64.4%) attended the follow-up appointment, while 136 (35.6%) did not follow the established action plan. On the other hand, in 34 persons whose clinical decision did not include referral to outpatient mental health services (11 patients were absconds prior to clinical decision from hospital emergency service and 23 patients were referral to primary care), these services were accessed by other referral routes. In the 6 months follow up, a total of 280 persons (50.5%) had any contact with a public mental health facility, while 274 persons (49.5%) did not. Finally, it should be noted that 129 people had a relapse of suicidal behavior in the subsequent 6 months (23.3%), compared to 425 who did not (76.7%) (Figure 2).

### Factors associated with the repetition of suicidal behavior

Stepwise logistic regression using forward elimination ended with 3 variables after 5 iterations. The final model included age, number of lifetime suicide attempts and any contact with a mental health center, in the 6 months following suicidal behavior. The model was statistically significant, χ^2^(4) = 148.13, *p* < 0.001. The model explained 32.8% (Nagelkerke R2) of the variance in reattempted suicide within 6 months and correctly classified 80.8% of the cases. The number of lifetime suicide attempts (adjusted odds ratio = 1.21, CI 1.148–1.270, *p* < 0.001), was associated with an increased likelihood of reattempting suicide. However, no contact with a mental health center within 6 months of emergency care (adjusted odds ratio = 0.395, CI 0.250–0.625, *p* < 0.001) and increasing age (adjusted odds ratio = 0.985, CI 0.972–0.999, *p* = 0.044) were associated with a decreased likelihood of reattempting suicide (Table 2).

**Table 2:** Variables associated with suicide reattempt in the 6 months after suicidal behavior attended by out-of-hospital emergency services in multiple logistic regression.

Dependent variable = Reattempted suicide within 6 months	Coefficient	Odds ratio	95% CI	*p*
Number of lifetime suicide attempts	0.188	1.207	1.148–1.270	**<0.001**
Any contact with a public mental health facility in the 6 months following emergency care	−0.928	0.395	0.250–0.625	**<0.001**
Age	−0.015	0.985	0.972–0.999	**0.044**
Constant	−0.991	0.371	0.000	**0.004**

Nagelkerke R squared: 0.320. Hosmer-Lemeshow test = 0.155. Bold values indicate significant results.

CI, Confidence Interval.

## Discussion

The aim of this study was to analyze the care pathway of patients seeking help in out-of-hospital emergency services for suicidal behavior, and to identify variables associated with the repetition of suicidal behavior. To our knowledge, there are no published studies in Spain that include information from two complementary emergency care services to trace the care pathway in public health services. These results are very useful for obtaining a global and holistic view of the health care system and the care of people with suicidal behavior problems.

Although our study is developed in one province of Spain (Malaga), our results can be generalized to the rest of Spain. We must take into account that Spanish National Health Service provides universal coverage and free access to health care for the population in Spain on a tax-based funding, covering 99.1% of the population (22). So, we are confident that any person with suicidal behavior that call an emergency line as 061 or 112, will be transfer to the public health hospitals of Malaga and this procedure is similar in the rest of Spain.

### Description of the sample

Since out-of-hospital emergency services attend to all types of injuries requiring urgent attention, these are in most cases of a physical nature, with suicide-related injuries accounting for only 1%. These results are in line with our previous studies (9, 23–25) and also with other studies carried out elsewhere (26–29) so our results are comparable to those carried out, in similar settings, elsewhere in the world.

Regarding the previous suicide attempts attended in public health facilities, first of all we would like to highlight that nearly half of the sample (44.2%) had no other suicide attempts, while the rest presented at least one. In this line, Vázquez-Lima et al. (25), in a study conducted in the Galician Hospital Emergency Department, found that previous suicide attempts were a precedent present in almost half of the patients who committed suicide. This is in line with our results, which slightly more than half of the sample had some previous suicide attempt attended by public health services.

### Description of health care

The majority of patients who are seen for suicidal behavior in out-of-hospital emergency services are transferred to the emergency services of their referral hospital (96%). This result is higher than that found by Duncan et al. (26), in which they indicate that 73.9% of people seen for suicidal behavior are transferred to the hospital. One explanation for the high percentage of people taken to the hospital by the mt-PCES is that this professionals are responsible for attending people at risk of suicide and decide to take them to the hospital to be seen by a mental health professional. According to Blanco-Sánchez et al. (30) mobile teams consider that their work is focused on attending to emergencies quickly and transferring patients to the hospital where they can be evaluated more accurately and mental health professionals can make the relevant decisions.

One result to highlight is that 82% of this group of patients is discharged home, once they have been treated in the hospital emergency services. This information is very interesting, especially for out-of-hospital emergency services, since they do not know what happens to the patients once they have been transferred to the hospitals. As they themselves have stated (30), these professionals carry out their actions quickly in life-threatening situations, and often avoid assessing in depth the intentionality of the behavior and its level of severity. They consider that it is the hospital care teams that can make a more adequate assessment of suicide risk (30). Therefore, when patients are assessed for suicide risk in a more comprehensive manner in the hospital, it could be concluded that the risk of completed suicide is unlikely and this would explain why many of the people referred for suicidal behavior by out-of-hospital emergency services are finally discharged by the hospital once their physical symptoms have been controlled. Another reason behind the low number of admissions to the mental health inpatient unit could be the organization of mental health care in Spain. The care model for people with suicide and mental health problems is mainly outpatient and community-based. Hospital admissions for a suicide attempt are restricted to those who have little social and/or family support or are seriously ill patients who are often involuntarily admitted. Only patients with a higher level of social issues (no accommodation, money problem) or less support in the community but moderate risk may be admitted. This result could be due to the design of our healthcare system and it may be the reason behind the low number of admissions to the mental health inpatient unit.

It has also been observed that 70% of the sample are referred to a mental health facility after emergency care, either to an outpatient mental health center or are admitted to the mental health inpatient unit. Of these, 30% are referred to their primary care physician.

In addition, of the 382 cases referred to mental health, the percentage of patients that have been attended in a mental health center at least once 6 months later was 51.4%, indicating that almost another 50% had not been seen by their mental health team. This figure is lower than that found by Soriano et al. (31), who reported that 58.7% were in follow-up in mental health services. Although there are several articles that analyze the use of health services prior to completed suicide (11, 32, 33), we are unaware of any studies that examine the use of services prospectively, once suicidal behavior has occurred, as we have done in our analysis.

Last but not least, it should be noted that almost 6% of those transferred leave the hospital emergency services voluntarily, before completing the care circuit in that service. One of the reasons for this could be the time the patient sometimes has to wait, either because of the time needed to obtain the results of complementary tests, or because of the wait for the specialist or simply due to these services being overburdened. In line with this observation, Ferreira et al. (27) reported that patients with suicidal behavior seen in emergency services have to wait longer than patients with other conditions. Another reason could be that some people with suicidal behavior have not come to the health services voluntarily, but are often transferred against their will, and therefore, if they see a possibility, they leave the hospital without completing the care circuit.

Thus, in light of these results, we can see that there is a group of patients who, once discharged from emergency services are lost from the public health system, together with those referred to primary care and those who leave emergency services. Although we know that some patients may be receiving private care and this information is therefore not available to us, because the public health system in Spain covers 99.2% of the population, we assume that the majority are receiving care through the public system. According to Riblet et al. (34), the low participation of patients in follow-up may be due to the stigma associated with psychiatric care for suicidal behavior, which complicates and hinders the treatment of these individuals.

To prevent this from happening, in Malaga (Spain), the public Mental Health Unit of the University Regional Hospital, where this research was carried out, has implemented a protocol called “Suicide Code,” which consists of follow-up through telephone calls to people discharged from emergency services who were treated for a suicide attempt, in order to guarantee attendance at subsequent mental health appointments.

### Factors associated with the repetition of suicidal behavior

To prevent, it is important to understand the factors associated with repetition of the behavior. Accordingly, this was our second objective. In this regard, 70% did not present repetition of suicidal behavior in the 6 months following emergency care.

The model obtained after regression analysis explains 32% of the variability and includes 3 variables, of which, as expected, the number of lifetime suicide attempts would be a risk factor that increases the likelihood that suicidal behavior will be repeated, as has been shown in various studies and meta-analyses (35–37).

Subsequent no contact with mental health center within 6 months of emergency care has been associated with a decreased likelihood of the person making a new suicide attempt. We believe that this finding may be related to the fact that patients referred to an outpatient mental health service are more severe and they present a higher risk of suicide attempt than those who have not been referred. Generally, this latter group is composed of people with suicidal ideation and people who engage in low-lethality suicidal behaviors, which could lead to a lower long-term risk of repeating suicidal behavior. As we are not analyzing active follow-up in mental health services we cannot compare our results with other studies that analyses active follow-up as the one developed by Inagaki et al. (38) and Duncan et al. (26) who concluded that active follow-up of people seen in emergency services reduces the risk of a new suicide attempt in the following 6 months. Further studies in this line are needed to be developed.

### On the classification of suicidal behavior calls

Although some articles based on the coding and classification of suicidal behavior cases have been questioned for their lack of reliability and consequently under- or over-recording of cases (39), in our study, we applied strict criteria to identify the calls as suicidal behavior, since we took into account both the clinical judgment of the out-of-hospital emergency doctor attending the patient *in situ* and the criteria of the operator responding to the telephone call at the 061 ECC. However, we are aware that a study carried out in our setting concluded that the classification system used in the 061 ECC has a sensitivity of 44.8% for detecting cases of suicidal behavior (16). Following on from this reflection, Anderson et al. (40) state that little training is available on how visits for suicidal behavior should be documented and that research that relies on international disease classification codes included in medical records to study suicide sometimes significantly underestimates cases of suicide attempts and suicidal ideation. Furthermore, in a recently published systematic review, we concluded that each out-of-hospital emergency service uses a different classification, making it difficult to make valid and reliable comparisons of the data collected in these care settings (41).

## Limitations

One of the main limitations of our study is that we used secondary data sources from routine clinical practice. This may affect the quality of the information analyzed, especially in the case of suicidal behavior. Nevertheless, the collection of information from the clinical records was comprehensive, and quality control of the data was performed. Another related limitation is that only those variables included in clinical practice could be analyzed, and therefore other variables that could be considered of interest are not available. However, the review of digitalized medical records to collect patient information is a method increasingly used in research on suicidal behavior and health services use, since most of these people come to health services to seek care and their calls are recorded. Another problem related to the reliability of the information is associated with the coding of suicidal behavior and specifically with the assessment of intentionality. A final limitation is that we only analyzed patients attended by mt-PCES professionals. Therefore, those cases of individuals who go directly to hospital emergency services were not analyzed, and the results cannot be generalized to these patients.

In conclusion, we believe that selective suicide prevention initiatives should be designed to target the population at risk of suicide, especially those receiving both out-of-hospital and in-hospital emergency services, since a better understanding of the characteristics and needs of this group would enable the development of strategies to improve the quality of care currently provided, especially the follow-up after a suicidal crisis identified in emergency services. In addition, better coordination between the two levels of care would allow a more appropriate approach to the needs of this group of patients.

## Data availability statement

The original contributions presented in the study are included in the article/supplementary material, further inquiries can be directed to the corresponding author.

## Ethics statement

The studies involving humans were approved by Ethics and Research Committee of Northeastern Malaga. The studies were conducted in accordance with the local legislation and institutional requirements. Written informed consent for participation was not required from the participants or the participants' legal guardians/next of kin in accordance with the national legislation and institutional requirements.

## Author contributions

JR-M, CG, AM-G, PC-J, JG-P, and BM-K contributed to conception and design of the study. JR-M and CG organized the database. CG performed the statistical analysis. JR-M, CG, and BM-K wrote the first draft of the manuscript. All authors contributed to the article and approved the submitted version.

## Funding

This study was funded by the Fundación Progreso y Salud (Junta de Andalucía). Number: AP-0226-2019; by “Línea Acciones Especiales” and “Ayudas para publicación en acceso abierto del II Plan Propio de Investigación y Transferencia de la Universidad de Málaga, 2023”.

## Conflict of interest

The authors declare that the research was conducted in the absence of any commercial or financial relationships that could be construed as a potential conflict of interest.

## Publisher’s note

All claims expressed in this article are solely those of the authors and do not necessarily represent those of their affiliated organizations, or those of the publisher, the editors and the reviewers. Any product that may be evaluated in this article, or claim that may be made by its manufacturer, is not guaranteed or endorsed by the publisher.
